# Controlled Immobilization Strategies to Probe Short Hyaluronan-Protein Interactions

**DOI:** 10.1038/srep21608

**Published:** 2016-02-17

**Authors:** Burcu Baykal Minsky, Christiane H. Antoni, Heike Boehm

**Affiliations:** 1 Department of New Materials and Biosystems, Max Planck Institute for Intelligent Systems, Heisenbergstr. 3, D-70569 Stuttgart, Germany; 2 Department of Biophysical Chemistry, University of Heidelberg, INF 253, D-69120 Heidelberg, Germany; 3 CSF Biomaterials and Cellular Biophysics, Max Planck Institute for Intelligent Systems.

## Abstract

Well-controlled grafting of small hyaluronan oligosaccharides (sHA) enables novel approaches to investigate biological processes such as angiogenesis, immune reactions and cancer metastasis. We develop two strategies for covalent attachment of sHA, a fast high-density adsorption and a two-layer system that allows tuning the density and mode of immobilization. We monitored the sHA adlayer formation and subsequent macromolecular interactions by label-free quartz crystal microbalance with dissipation (QCM-D). The modified surfaces are inert to unspecific protein adsorption, and yet retain the specific binding capacity of sHA. Thus they are an ideal tool to study the interactions of hyaluronan-binding proteins and short hyaluronan molecules as demonstrated by the specific recognition of LYVE-1 and aggrecan. Both hyaladherins recognize sHA and the binding is independent to the presence of the reducing end.

Hydrophilicity, biocompatibility, biodegradability and animal independent sources with high purity are desirable properties for a material in bioengineering that are fulfilled by the biopolymer hyaluronan (hyaluronic acid, HA)^1^. Additionally, HA can easily be chemically modified to facilitate covalent attachments^2^, an essential property to generate hydrogels^3^ and immobilize it onto surfaces to control cell adhesion and protein adsorption^4^,^5^. HA can control not only the physicochemical properties of synthetic surfaces, but also induce specific biological responses through HA/HA-receptor interactions. Consequently, tuning the material interfaces by tailoring chemical, mechanical and biological properties of HA facilitates novel approaches and makes it an attractive candidate in bioengineering^6^. Therefore, novel tools are required to analyze specific interactions of HA with the diverse range of HA-binding proteins present in biological environments to fully utilize the unique properties of HA.

HA is a negatively charged linear polysaccharide consisting of repeating disaccharide units of glucuronic acid and N-acetylglucosamine at the reducing end. Chemical modifications of HA, such as functionalization at the reducing end with a thiol group, enable direct immobilization on gold surfaces, which have been successfully used as sensors for HA degrading species, such as enzymes and reactive oxygen species^7^. Immobilization strategies of HA are also aimed towards generating biomimetic systems such as employing end-biotinylated HA linked to streptavidin containing supported lipid bilayers (SLB)^8^ to investigate interactions between HA and hyaladherins^9^, immobilizing biotinylated HA to investigate selective inhibitors for CD44^10^, using HA and chitosan for layer by layer assembly for intraocular lens surface coating^11^ and grafting HA to glass via NHS chemistry to investigate cell adhesion^12^.

HA is predominantly found in the extracellular matrix of soft connective tissues like the skin, synovium, cartilage and the vitreous humour in all vertebrates^13^. The biological function of hyaluronan has not only been ascribed to its association with different HA binding proteins (hyaladherins)^14^, but also to its size/length. Low molecular weight HA oligosaccharides (LMW HA or sHA) are associated with angiogenesis^15^,^16^, especially lymphangiogenesis^17^, inflammation^18^ as well as tumor invasion and lymph node metastasis^18^,^19^. Different mechanisms postulated for the size dependent HA bioactivity include competition of sHA with high molecular weight HA on cellular receptors^18^ and inability of sHA clustering of HA-cell receptors. However, another striking difference between low and high molecular weight hyaluronan lies in the higher ratio of terminal groups in the low molecular weight samples with the same overall mass. Particularly, the unique chemical properties of the reducing ends have been implicated in the specific recognition of hyaladherins^20^.

In this study, we develop two strategies for controlled sHA presentation that allow us to (1) study the specific recognition of the reducing end of sHA molecules and to (2) tune the density of sHA on an otherwise inert background. This enables us to compare the immobilization dynamics and bioactivity of differently functionalized sHA molecules. We monitor the specific interactions of immobilized sHA by a quartz-crystal microbalance with dissipation monitoring (QCM-D) for facile and sensitive analysis of surface adsorption and interactions in real time^21^. QCM-D enables not only the label-free analysis of adsorbed wet masses, but also gives insight into the viscoelastic properties of the formed adlayers and their rearrangement, which is especially important in the highly hydrated HA-supramolecular systems.

## Results and Discussion

### Homogeneous Immobilization of sHA via the Reducing End

**Figure 1:**
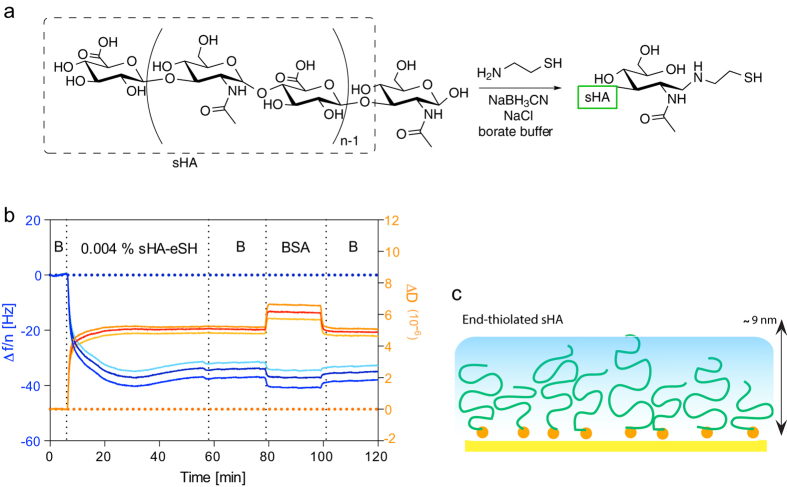
(**a**) Thiol-end modification scheme for sHA (sHA-eSH). (**b**) QCM-D response, i.e., changes in frequency (Δf/n, shades of blue) and dissipation (D, shades of orange) induced by grafting sHA-eSH and subsequently introducing 0.5% BSA to investigate unspecific adsorption. (**c**) Proposed architecture of sHA-eSH immobilized on the gold sensor surface. (B: refers to buffer, PBS).

The high-density presentation of sHA was achieved with a straightforward and fast method by modifying the reducing end^7^ of sHA with a short thiol linker (sHA-eSH) (Fig. 1a) and its subsquent immobilization on gold surfaces. The saturation of the gold QCM-D sensor surface with sHA-eSH was almost complete within 10 minutes (Fig. 1b) and the following washing steps with buffer did not induce any change in frequency, indicating strong binding between the sHA-eSH and the gold sensor, Introducing unmodified sHA on the gold QCM-D sensor did not show a significant frequency change, which confirms formation of a dense adlayer by sHA-eSH, but not by unmodified sHA (Supporting Information, Figure S1). Additionally, when BSA is washed over sHA-eSH modified surfaces, BSA doesn’t induce any significant change in frequency apart from its typical buffer effect^22^, which is related to the viscosity difference of BSA solution. Thus, sHA-eSH modified surfaces are inert towards unspecific protein adsorption. This effect was also compared with poly(l-lysine)-graft-poly(ethylene glycol) (PLL-g-PEG), a widely used copolymer in biomolecular interfaces to create an inert background and control protein adsorption^23^,^24^. PLL-g-PEG was grafted on silica QCM-D sensor in flow mode (Supporting Information, Figure S2) and layer formation is quite stable with a frequency change of Δf_7_ = 28.7 ± 0.18 Hz and dissipation change of ΔD_7_ = 2.3 ± 0.05 × 10^−6^ upon adsorption. The adsorbed layer is less viscoelastic compared to OEG/sHA adlayer, as indicated by the smaller change in dissipation. BSA wash did not induce significant frequency or dissipation change on PLL-g-PEG after buffer wash steps, similar to the behavior of BSA on OEG/sHA layers.

Immobilization of sHA-eSH enables a fast coverage of gold surfaces with the highest possible surface-coverage achievable in such a grafting-to approach in which the full chain is immobilized directly to the surface. However the grafting density can only be controlled indirectly. The gold surface offers a large number of binding sites for sHA-eSH and this does not limit the grafting density. Thus, the surface coverage is determined by the polymer properties of sHA, such as its high degree of hydration, flexibility and its length. The average contour length (*L*_*c*_) of sHA used in the experiments is approximately 24 nm with a radius of gyration (*R*_*g*_) of ca. 5.3 nm^25^,^26^. In contrast, when the QCM-D curves of the sHA-eSH layers are fitted based on the “extended viscoelastic model” and their thickness was determined to be about 9 nm. This indicates the limited stretching of sHA-eSH chains immobilized at this grafting density. Additionally, an optical technique, two wavelength multi parametric surface plasmon resonance (MP-SPR), was applied to determine the layer thickness *(vide infra)*. In good agreement with the QCM-D modeling, the thickness obtained using MP-SPR was 9.4 nm (Table 1). Simultaneously, the refractive index of the adlayer is determined (Table 1) and the adsorbed dry surface mass can be calculated using the specific refractive index increment of HA, dn/dc of 0.15 cm^3^/g^27^. Thus, the immobilization of sHA-eSH onto a gold surface leads to a surface mass of 255 ng/cm^2^. Assuming a spherical shape corresponding to the radius of gyration of HA for calculation purposes, the average minimal spacing of hyaluronan molecules would correspond to about 2 nm, as hyaluronan density decreases from the surface towards the solution^28^,^29^.

### Two-Layer System for Adjustable Immobilization Density

**Figure 2:**
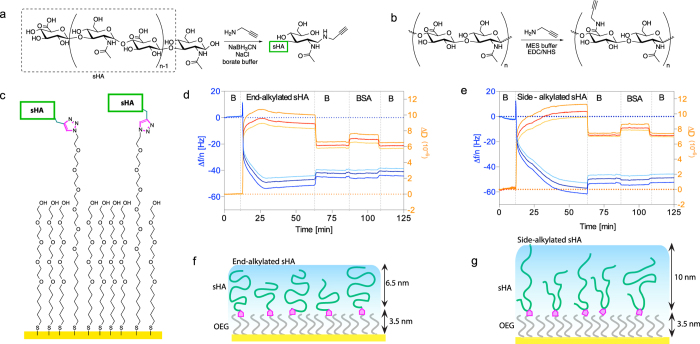
Alkyne modifications on sHA (**a**) at the reducing end (end-alkylated) and (**b**) within the chain on carboxyl groups (side-alkylated). (**c**) Mix SAMS are formed by coadsorbing EG_6_sHA conjugate with EG_3_OH. QCM-D responses, (Δf/n, shades of blue) and dissipation (D, shades of orange) shifts, for (**d**) OEG/end-alkyl sHA and (**e**) OEG/side-alkyl sHA at 67% EG_6_sHA ratio, and passivation strength was evaluated using 0.5% BSA (B: refers to buffer, PBS). Proposed immobilization profile for (**f**) OEG/end-alkylated sHA and (**g**) OEG/side-alkylated sHA.

In order to control the density of immobilized sHA molecules on a surface, we establish an adjustable well-defined two-layer system. For this purpose, sHA was alkylated with a linker bearing an alkyne group at the reducing end (end-alkylated, Fig. 2a). This alkyne group was conjugated with oligo(ethylene glycols)-alkanethiols (OEG-alkanethiols) bearing azide terminal groups (EG_6_N_3_:HS-(CH_2_)_11_-EG_6_-N_3_) via copper catalyzed alkyne/azide click chemistry. The EG_3_OH was coadsorbed with sHA functionalized OEGs to adjust the density of sHA on the surface (Fig. 2c). Using EG_3_OH as a diluent, not only provides adjusting the density of sHA, but also ensures an inert surface against both protein adsorptions like BSA and unfunctionalized sHA. The QCM-D profile of EG_3_OH (Fig. 2d) coadsorption with 67 mole percent OEG/end-alkylated HA (Fig. 2e) induces frequency and dissipation changes of Δf_7_ = 41.9 ± 0.12 Hz and ΔD_7_ = 6.2 ± 0.01 × 10^−6^ after about 10 minutes. The resulting OEG/end-alkylated sHA surface was inert towards unspecific protein adsorption, as passing BSA over the sHA adlayers did not lead to any notable frequency or dissipation change (Fig. 2d). Compared to sHA-eSH grafting, OEG/end-alkylated (Fig. 2f) yields a smaller sHA layer thickness of ca. 6.5 nm from fitting the QCM-D data, which could be due to lower grafting densities.

### Covalent Immobilization of sHA via the Carboxyl Group

The bioactivity of small hyaluronan molecules is distinctly different from hyaluronan polymers with a length of up to several micrometers and also varies considerably in the density of reducing-end groups. In the immobilization methods described so far, the reducing-end was utilized to attach sHA molecules to the surface and is thus not accessible anymore. In order to immobilize sHA in a manner in which the reducing end would be available to participate in protein binding interactions, sHA was also alkylated with a linker bearing an alkyne group within the HA chain at carboxyl groups^30^ of the glucuronic acid repeating unit (side-alkylated, Fig. 2b). As explained before, alkyne groups were conjugated with EG_6_N_3_ via the copper catalyzed alkyne/azide click chemistry and EG_3_OH was coadsorbed with sHA functionalized OEGs to adjust the density of sHA on the surface (Fig. 2c). The QCM-D profiles of EG_3_OH coadsorption with 67 mole percent OEG/side-alkylated HA content (Fig. 2d) yields slightly higher values (Δf_7_ = 49.5 ± 0.09 Hz and ΔD_7_ = 7.2 ± 0.01 × 10^−6^) after washing with PBS. These surfaces were also inert towards unspecific protein adsorption, as passing BSA over the sHA adlayers did not lead to any notable frequency or dissipation change (Fig. 2e). Corresponding to the slightly higher changes in adsorbed mass, OEG/side-alkylated sHA layer leads to thickness values of about 10 nm (Fig. 2g).

### Comparison of the Different sHA Layers

The immobilization of sHA leads to splitting of QCM-D overtones, which demonstrates formation of soft and highly hydrated viscoelastic films^8^. In addition, the rigidity profiles of immobilized sHA are evaluated from D/f plots, which provide a relative measure for the changes in the dissipation per unit added mass (Supporting information, Figure S3). The adsorption profile clearly indicates a dynamic profile especially for OEG/side alkylated sHA. In addition, water contact angle (WCA) measurements showed formation of highly hydrophilic surfaces when gold-coated surfaces (WCA 69°) were grafted with different ratios of OEG/sHA (WCA < 20°), while the control EG_3_OH/EG_6_N_3_ surfaces exhibited WCA 48°. On the contrary, PLL-g-PEG coated surfaces exhibited WCA of 27° on the activated glass surfaces (untreated glass WCA 64°).

In comparison to QCM-D analysis, two-wavelength multi-parametric surface plasmon resonance (MP-SPR) was applied to determine the thicknesses of the sHA adlayers. MP-SPR is an optical technique and measures the changes in the refractive index on the sensor surface, which is directly related to the mass. Therefore, MP-SPR is sensitive to the dry mass as opposed to wet mass including hydrodynamically coupled water obtained from QCM-D. In MP-SPR technique, measurements are performed using two wavelengths (670 and 785 nm); therefore thicknesses and the refractive indices can be obtained simultaneously^31^,^32^. The analysis was performed for EG_3_OH/EG_6_N_3,_ sHA-eSH and OEG/side&end-alkylated sHA and the data evaluation was performed using BioNavis LayerSolver software (Supporting Information, Figure S4 and S5). The results for the thickness values and the refractive indices are summarized in Table 1. The thickness values obtained from MP-SPR are in well agreement with the calculated thickness values from the QCM-D experiments. OEG/side-alkylated sHA has the highest thickness value, followed by the OEG/end-alkylated sHA and end-thiolated sHA.

All three methods of immobilization lead to sHA molecules stretched compared to their radius of gyration *R*_*g*_ *(5.3 nm),* but remain a lot smaller than their contour length *L*_*c*_ *(24 nm)*. This limited stretching could be explained by the following: the grafting density of the polymer determines the architecture of the film, where at low densities the polymer may bind to the surface in its native coiled form, whereas at high densities, chains start to overlap and form extended brushes^33^. However, sHA may not be treated as a simple brush, because it carries a high negative charge density and does not have a monodisperse size distribution. A study put forward by de Vos and Leermakers, using numerical self-consistent field model, showed that polydispersity strongly influences the average stretching in the brush and it decreases with increasing polydispersity^34^.

### Interaction of Immobilized sHA with Hyaladherins

**Figure 3:**
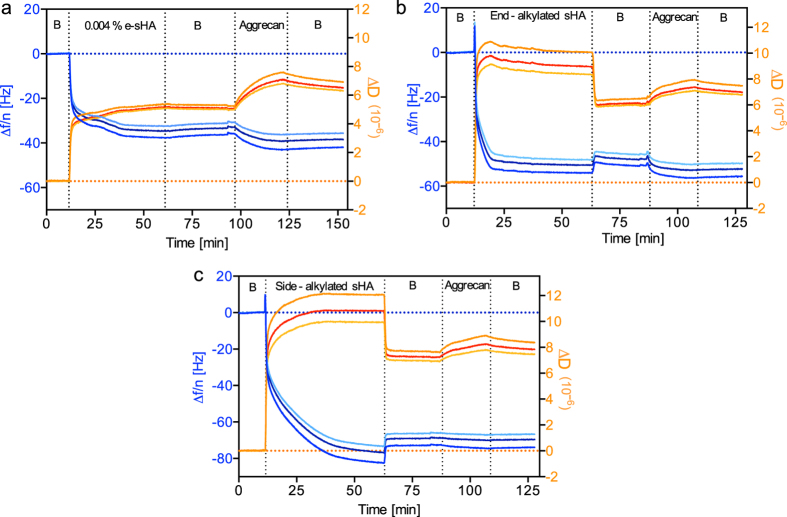
Aggrecan binding to the immobilized (**a**) sHA-eSH, (**b**) OEG/end-alkylated sHA and (**c**) OEG/side-alkylated sHA both at a ratio of 67% EG_6_sHA. (B: refers to buffer,PBS).

HA organizes, stabilizes and remodels the extracellular matrix (ECM) with its associated hyaladherins, and this dynamic network controls the interactions of cells with their extracellular environment. Therefore, we evaluated the bioactivity of sHA immobilized by the different immobilization methods focusing on the interaction with the ECM proteoglycan, aggrecan and, a sHA sensitive cell receptor, lymphatic vessel endothelial hyaluronan receptor 1 (LYVE-1)^17^. In contrast to protein-protein interactions, most carbohydrate-protein domain interactions are mostly mediated by very shallow, weakly binding protein binding grooves and K_D_ values for HA-hyaladherin interactions are in the range of 30–60 μM^35^. Aggrecan increases load bearing properties in tissues, such as in cartilage^36^, induces swelling of HA chains^37^ and also behaves as a protective agent for oxidative stress in neurons^38^. Our QCM-D results show that all three types of immobilized sHA can interact with aggrecan (Fig. 3a–c). The surfaces functionalized with oligo(ethylene) glycol (EG_3_OH) did not show aggrecan binding (Supporting Information, Figure S6a), thus indicating aggrecan binding occurs specifically to the sHA. Even though dissipation shifts were observed in all three surfaces, the prominent frequency change was observed for sHA-eSH and OEG/end-alkylated sHA, but not for OEG/side-alkylated sHA. The binding decreased slightly with lower densities of sHA on the two-layer systems, but remained higher on the end-alkylated sHA compared to the side-alkylated sHA (Supporting Information, Figure S7). This qualitative difference clearly indicates that aggrecan does not recognize the reducing end as expected.

**Figure 4:**
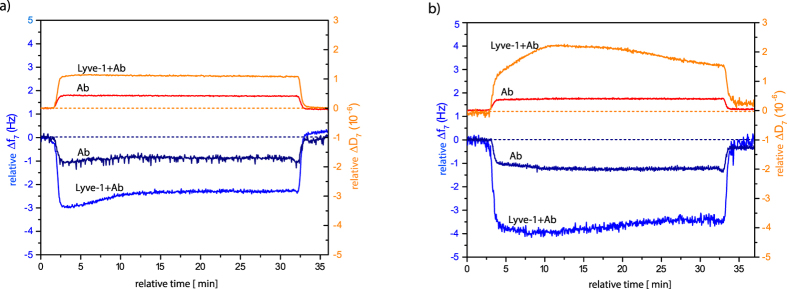
QCM-D profile of antibody conjugated LYVE-1 interaction with the 33% (**a**) side-alkylated and (**b**) end-alkylated sHA surface. For easier comparison, all values were set to zero after functionalization with OEG/sHA.

LYVE-1 is a membrane protein and the main HA receptor expressed on lymphatic endothelial cells^39^. In particular, LYVE-1 is linked to lymphangiogenesis through interactions with sHA^17^. Therefore, we probed the binding capacity of the OEG-azide/sHA-alkyne layers towards the HA binding domain of LYVE-1 (Fig. 4). The ectodomain of LYVE-1 is 30 kDa, which is very small to be detected on the QCM-D sensor. Therefore, it is conjugated with its specific antibody to increase the QCM-D response. In the presence of the LYVE-1-antibody complex, the frequency shift increased on both alkylated OEG-sHA surfaces compared to the only antibody binding (Figure 4 and Supporting Information, Figure S8). In addition, LYVE-1-antibody complex does not interact with EG_3_OH surfaces (Supporting Information, Figure S6b). Similar to the aggrecan binding, the reducing end does not seem to be involved in LYVE-1/sHA interactions. Even though a QCM-D response was detectable upon addition of the LYVE-1-antibody complex, the binding was reversible, and the frequency and dissipation values leveled back to initial values immediately after the washing step. This result might suggest that the binding has high off-rates or that other yet unidentified co-factors are necessary for stronger binding affinities.

In summary, we achieved a high surface coverage of covalently immobilized sHA molecules on gold surfaces by introducing a thiol group at the reducing end of sHA. We compared this layer with tunable adlayers based on self-assembled oligo-ethylenglycol layers of EG_3_OH mixed with adjustable ratios of EG_6_HA, which had been covalently linked via click chemistry. All of the resulting sHA layers prevent unspecific adsorption while maintaining the ability to be specifically recognized by hyaladherins. Immobilization of sHA via the reducing end or within the chain further allows probing the importance of the reducing end as a vital difference between hyaluronan molecules of different lengths. Interestingly, the specific recognition by both aggrecan and LYVE-1 did not dependent on the specific recognition of the reducing end of sHA. These different immobilization methods pave the way for dual-functionalized surfaces to investigate more complex systems and enhance probing a wide range of specific interaction mechanisms of hyaluronan and its specific binding proteins.

### Materials

Hyaluronic acid sodium salt (HA, research grade), M_r_ = 10 kDa, was purchased from LifeCore Biomedical (Chaska, MN, USA). Cysteamine hydrochloride, sodium cyanoborohydride, sodium chloride, dithiothreitol (DTT), sodium tetraborate, 2-(*N*-morpholino)-ethanesulfonic acid (MES), *N*-(3-dimethylaminopropyl)-*N’*-ethylcarbodiimide (EDC*HCl), *N*-hydroxysuccinimide (NHS), CuSO_4_, ascorbic acid, TRIS and propargylamine were all obtained from Sigma Aldrich (Taufkirchen, Germany). Dialysis tubes (MWCO: 2000 and 3500 Da) were purchased from Carl Roth (Karlsruhe, Germany). Alkanethiols carrying oligo(ethylene glycol) (OEG) with a hydroxyl termini (EG_3_OH: HS-(CH)_11-_EG_3_-OH) and azide termini (EG_6_N_3_: HS-(CH_2_)_11_-EG_6_-N_3_) for surface functionalization were purchased from ProChimia (Sopot, Poland). Human recombinant LYVE-1 and aggrecan were purchased from R&D Systems and Sigma, respectively. PLL-g-PEG: PLL(20)-g[3.5]- PEG(5) = PLL(20 kDa) grafted with PEG(5 kDa) was obtained from SuSos (Dübendorf, Switzerland).

## Methods

### Synthesis of End-Thiolated sHA (sHA-eSH)

Functionalization of sHA was carried out according to the protocol established by Lee *et al.* at the reducing N-acetylglucosamine unit^7^. Therefore, sHA (100 mg) and cysteamine hydrochloride (120 mg, 100 mM) were dissolved for 2 hrs at room temperature in NaCl (400 mM) containing borate buffer (20 mL, 100 mM, pH 8.5). Sodium cyanoborohydride (251 mg, 200 mM) was added to the reaction mixture and stirred for 5 days at 40 °C. The resulting mixture was incubated with DTT for 2 hrs at 40 °C and dialyzed (MWCO: 2000 Da) against NaCl/HCl/Milli-Q water for 2 hrs and then against HCl/Milli-Q water for 2 days. The thiolated sHA was recovered by freeze-drying and verified by the Ellman’s assay^40^ and stored at −80 °C.

### Synthesis of Alkyne End-functionalized sHA

The same protocol for functionalization of the reducing end^7^ was adapted to introduce an alkyl group. Initially, sHA (100 mg) and propargylamine (670 μL, 576 mg, 523 mM) were dissolved for 2 hrs at room temperature in NaCl (400 mM) containing borate buffer (20 mL, 100 mM, pH 8.5). Sodium cyanoborohydride (251 mg, 200 mM) was added to the reaction mixture and stirred for 5 days at 40 °C. The reaction solution was dialyzed (MWCO: 2000 Da) against Milli-Q water for 2 days, lyophilized and stored at −80 °C.

### Synthesis of Alkyne-functionalized sHA

Functionalization of sHA within the chain was carried out following the protocol by Crescenzi *et al.*^30^ targeting the carboxyl groups. Thus, sHA (200 mg) was dissolved in MES buffer (8 mL, 50 mM, pH 4.0). EDC*HCl (287 mg, 187 mM), NHS (287 mg, 312 mM) and propargylamine (243 μL, 209 mg, 474 mM) are added to the solution. The reaction mixture was stirred at room temperature for 24 hrs. The solution was dialyzed (MWCO: 3500 Da) against a saturated NaCl solution for 1 day and against Milli-Q water for 5 days. The alkylated sHA was recovered by freeze-drying and stored at −80 °C. ^1^H-NMR (399.89 MHz, D_2_O): δ = 4.48 (dd, J = 7.8 Hz, J = 41.1 Hz, 2H, C*H*_2_-OH), 3.92–3.39 (m, 9 H, unhydroglucose unit of HA), 3.31 (t, J = 7.8 Hz, 1 H, C*H*-CH_2_OH), 2.88–2.84 (m, 0.6 H, C*H*_2_-C≡CH), 2.67–2.54 (m, 0.08H, CH_2_-C≡C*H*), 1.98 (s, 3H, NH-CO-C*H*_3_) ppm.

### Alkyne/azide Click Reaction and Surface Functionalization

Click reaction was performed using TRIS buffer (100 mM pH 8.5), ascorbic acid (100 mM), EG_6_N_3_ (100 μM) and alkylated sHA (ca. 100 μM) in the presence of 1 mM CuSO_4_ at room temperature for 1.5 hrs. EDTA (final concentration of 1 mM) was added to chelate copper to stop the reaction. The resultant EG_6_HA was aliquot and stored at −20 °C. Adlayers with different ratios were prepared by diluting EG_6_HA with EG_3_OH, and adjusting the total thiol concentration to 100 μM in the corresponding buffer.

### NMR

The verification of alkyne-modified sHA was carried out by ^1^H-NMR and ^1^H,^1^H-COSY-NMR spectroscopy with a Bruker Avance II 400 NMR and the software TopSpin 3.2 (Bruker Bio Spin Corporation, Billerica, MA, USA) was used for the analysis.

### Quartz Crystal Microbalance with Dissipation Monitoring (QCM-D)

QCM-D measures the change in resonance frequency (Δf), adsorbed mass including hydrodynamically coupled water, and dissipation (ΔD), softness/viscoelasticity of the adlayers, as a result of material adsorption on the surface of sensor crystals. QCM-D measurements were performed with the Omega Auto (Q-Sense AB), which includes a fully automated sample handling of four sensors in parallel. Each channel runs independently with separate automated Hamilton syringe pumps. The system was operated in flow mode with a flow rate of 20 μL/min at 23 °C. QCM-D data were collected at seven overtones (n = 1, 3, 5, 7, 9, 11, 13, corresponding to resonance frequencies of ~5, 15, 25, 35, 45, 55, 65 MHz). Changes in dissipation and normalized frequency, Δf = Δf_n_/n, of the fifth, seventh and ninth overtones (n = 5,7,9) are presented in the graphs. Error bars for individual measurements are calculated by averaging over the last five minutes of the buffer wash before adding the samples and the final buffer wash. The errors are the sum of both standard deviations^41^. Prior to experiments, gold-coated (QSX301; Q-Sense AB) and silica (QSX303; Q-Sense AB) QCM-D sensors were treated with O_2_ plasma at 150 watts, 0.4 mbar for 45 min. They were cleaned with 2% hellmanex solution and Milli-Q water upon completion of the experiment and blow-dried using nitrogen. Thickness calculation for the sHA layers was performed using QTools v3. Software (Q-Sense AB) using “extended viscoelastic model” and Voigt model^42^ and the grafted layers were treated as single layer. The initial layer densities for eSH-HA, OEG/end-alkylated sHA and OEG/end-alkylated sHA were estimated as 1100 kg/m^3^, 1200 kg/m^3^ and 1300 kg/m^3^, respectively.

### Multi-Parametric Surface Plasmon Resonance (MP-SPR)

The surface densities and thicknesses of the OEG and OEG/sHA layers were determined using MP-SPR Navi™ 200-L (BioNavis Ltd., Ylöjärvi, Finland) by measuring angular spectra simultaneously at 670 and 785* *nm. The measurements were performed at 20 °C using 10 μL/min flow rate, and PBS was used for running buffer and sample dilutions. The samples were adsorbed for 60 minutes to ensure SAM formation. The results were pretreated by MP-SPR Navi DataViewer v.4.2.3, TraceDrawer for BioNavis v.1.6 and LayerSolver v.1.0.2 evaluations. The Au-coated SPR sensors (BioNavis Ltd., Ylöjärvi, Finland) were used in the experiments and cleaned by oxidative treatment. The measurements were started immediately after sensor cleaning to ensure clean surface for the formation of self-assembly.

### Sample Preparation for Bioactivity Measurements

Aggrecan from bovine cartilage (Sigma) was dissolved in PBS under constant stirring at room temperature for 1 h, and 1 mg/mL aliquots stored in −20 °C, and it is diluted to 100 μg/mL for QCM-D experiments. Recombinant Human LYVE-1with a C-terminal 6-His tag (R&D Systems, USA) was diluted to 20 μg/mL using 150 mM NaCl, 25 mM TRIS buffer (pH 7.4) and was incubated with monoclonal anti-polyhistidine–alkaline phosphatase antibody (Sigma) with a 1:500 dilution for 1 h at room temperature prior to QCM-D experiments. The complete QCM-D experiments were carried out in the same type of buffer in which samples were dissolved.

### Surface Preparation for Contact Angle Measurements

Silicon wafers (1 cm^2^ pieces) were coated with 100* *nm gold using thermal evaporation method at evaporation rate 1 Å/s and pressure 10^−6^ mbar (Pfeiffer Vacuum Classic 500). Prior to gold coating, silicon wafers were soaked in piranha solution (3:1 v/v mixture of 30%H_2_O_2_ and H_2_SO_4_) for 1 hour at room temperature, thoroughly rinsed with Milli-Q water and finally dried using nitrogen stream.

### Contact Angle Measurements

Measurements were performed using contact angle measuring device OCAH 230 (DataPhysics Instruments GmbH, Flindern, Germany) applying sessile drop method. In brief, 1 μL of water was deposited on the surfaces and the image was captured using CCD camera. The contact angles from both left and right sides were evaluated using SCA20 software and multiple measurements were done on different spots. All measurements were performed under ambient conditions at relative humidity ca. 39%.

## Additional Information

**How to cite this article**: Minsky, B. B. *et al.* Controlled Immobilization Strategies to Probe Short Hyaluronan-Protein Interactions. *Sci. Rep.* **6**, 21608; doi: 10.1038/srep21608 (2016).

## Supplementary Material

Supplementary Information

## Figures and Tables

**Table 1:** The surface thickness/RI analysis obtained from MP-SPR study and comparison with thickness calculated from QCM-D for the complete adlayer including the OEG bottom layer.

Samples	Thickness QCM-D(nm)	Thickness MP-SPR(nm)	Refractive index MP-SPR (RIU)
EG_3_OH/ EG_6_N_3_^*a*^	3.5 ± 0.5	3.6 ± 0.1	1.45
sHA-eSH	9.4 ± 1.8	9.4 ± 0.5	1.37
OEG/side-alkyl sHA^*b*^	12.7 ± 0.6	13.6 ± 0.5	1.36
OEG/end-alkyl sHA^*c*^	10.7 ± 0.2	12.1 ± 0.5	1.38

Error bars represent the standard deviations from at least three measurements (QCM-D) and fitting uncertainty (MP-SPR). *a:* EG_6_N_3_ molar concentration is adjusted to 67% in the total SAM.

*b,c*: side and end-alkyl sHA molar concentration is adjusted to 67% in the total SAM.
