# Supplementary material for: Controlled Immobilization Strategies to Probe Short Hyaluronan-Protein Interactions
Source: Sci Rep. 2016 Feb 17;6:21608. doi: 10.1038/srep21608 (PMC4756360; doi:10.1038/srep21608)
Supplement: Supplementary Information [file srep21608-s1.pdf]

Supporting Information

Controlled Immobilization Strategies to Probe Short Hyaluronan-Protein Interactions

Burcu Baykal Minsky[a], Christiane H. Antoni[a], Heike Boehm[a,b*]
[a] Department of New Materials and Biosystems, Max Planck Institute for Intelligent Systems, Heisenbergstr. 3, D-70569 Stuttgart, Germany & Department of Biophysical Chemistry, University of Heidelberg, INF 253, D-69120 Heidelberg, Germany
[b] CSF Biomaterials and Cellular Biophysics, Max Planck Institute for Intelligent Systems
* E-mail: boehm@is.mpg.de

Supplemental Results and Discussion

Control experiment for the adsorption of sHA on bare gold surface. When sHA-eSH is introduced on the QCM-D sensor, the postulated adsorption mechanism occurs through thiol-gold binding. To test this hypothesis, we check if unmodified sHA would interact with the gold surface. Introducing unmodifed sHA induces an inital change of about 8 Hz and is nearly removed from the surface after washing leading to a final frequency change of 3 Hz (Figure S1). However, sHA-eSH leads to a 40 Hz response and washing does not affect the frequency value (Figure 1). Thus, this indicates formation of a dense adlayer by sHA-eSH, but not by unmodified sHA. In addition, sHA does not induced observable change of frequency after washing on the only EG_3OH functionalized surfaces.

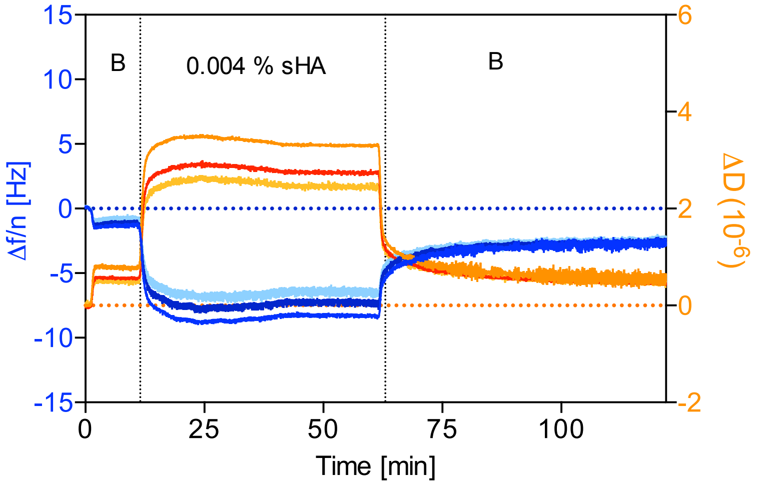

Figure S1. Unmodified sHA does not bind to gold QCM-D sensors and is easily washed of with a buffer (B) rinse.

Comparison of Antifouling Properties of sHA and PLL-g-PEG. As sHA functionalized surfaces were resistant to unspecific protein adsorption, we investigated PLL-g-PEG, which is a widely used polymer to create bioinert surfaces. QCM-D profile of BSA binding on the PLL-g-PEG coated silica surface exhibits a very similar trend for PLL-g-PEG and sHA functionalized surfaces.

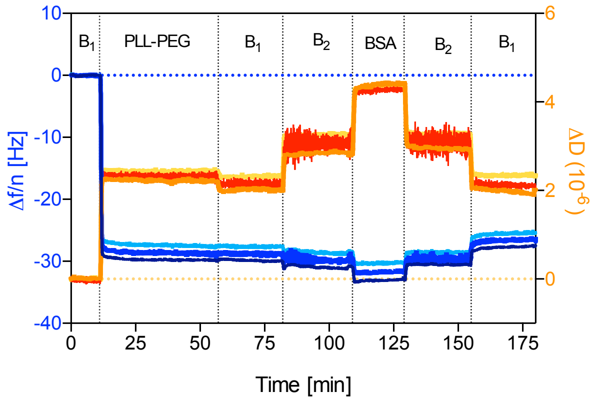

Figure S2. Adsorption of BSA on the PLL-g-PEG coated silica surface. PLL-g-PEG and BSA were dissolved in 10 mM HEPES buffer, pH 7.4 (B_1) and PBS (B_2), respectively. Introducing BSA to the system leads to a buffer effect but no binding after rinsing with buffer.

Comparison of adsorption profiles of different modification protocols of sHA. In order to evaluate the evolution of the rigidity of sHA films, $\Delta D_7/\Delta f_7$ is plotted against time (Figure S1). D/f plots provide a relative measure for the changes in the dissipation per unit added mass.[1] End-thiolated sHA exhibits a fast adsorption and quick saturation, which could be due to strong interactions between thiol groups and the gold surface. The most pronounced difference was seen for side-alkylated sHA adsorption, which exhibits a more dynamic profile and this could be a direct indication for the formation of a softer film. The sHA provided by LifeCore exhibits some degree of polydispersity, and side attachment could induce even higher apparent polydispersity, and subsequently larger variations in the density profile of the immobilized sHA,[2] which could lead to an altered hydration profile.

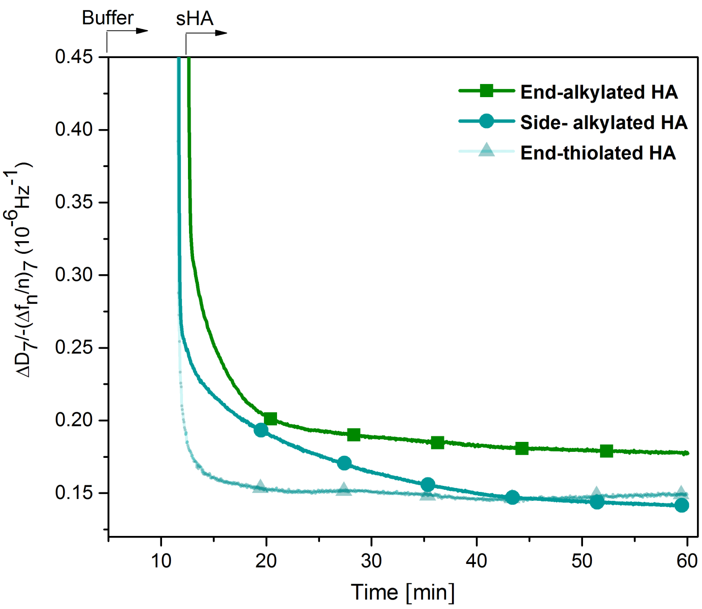

Figure S3. Rigidity profiles of differently immobilized sHA molecules evaluated by the ratio of dissipation changes to the normalized frequency changes during the adsorption process.

Multi-Parametric Surface Plasmon Resonance (MP-SPR) study to monitor thicknesses of the grafted sHA molecules on gold surfaces.

In order to validate the thickness values obtained from QCM-D for differently functionalized sHA layers, two-wavelength MP-SPR was employed. In this study, the samples were injected after 5 minutes of stable baseline after introducing the buffer (PBS) onto the gold sensors. The samples had different bulk refractive index compared to the background PBS. The bulk shift was corrected for using the PureKinetics™ software.

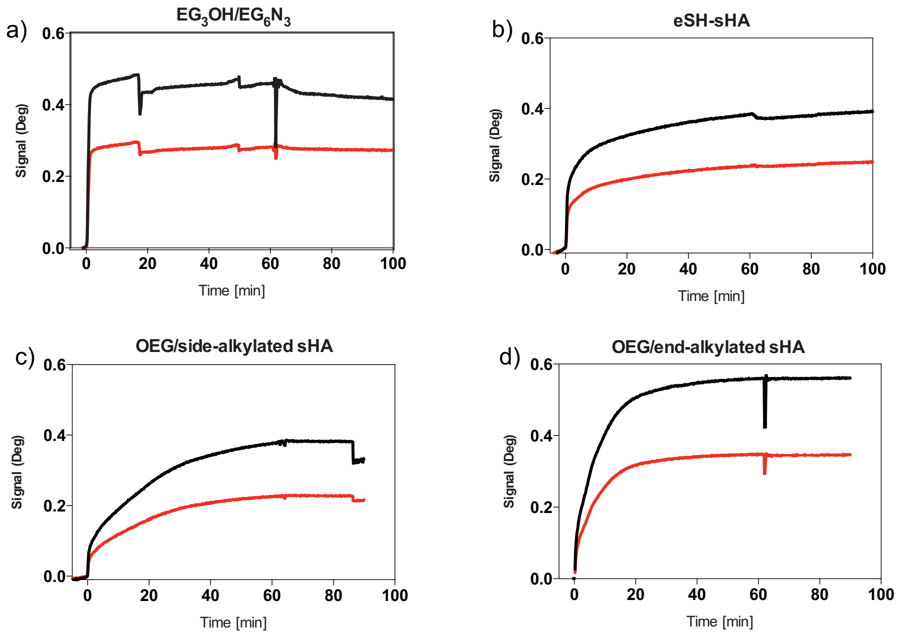

Figure S4. Angle-time sensorgrams of a) EG_3OH/EG_6N_3, b) eSH-sHA (0.04 mg/mL) c) OEG/side-alkylated sHA and d) OEG/end-alkylated sHA evaluated by PureKinetics analysis. (The EG_6N_3, and sHA content were adjusted to 67 mole percent). Black and red curves represent measurements at 670 nm and 785 nm, respectively.

In SPR, the shifts in the optical resonance properties of the sensor are correlated with the adsorbed materials and layer thickness. Applying two wavelengths in MP-SPR enables estimation of both refractive index and the thickness values by using Fresnel equations[3], which is implemented in the BioNavis LayerSolver software. The Figure S4 shows an example fitting analysis for OEG/side-alkylated sHA before and after introducing the sample.

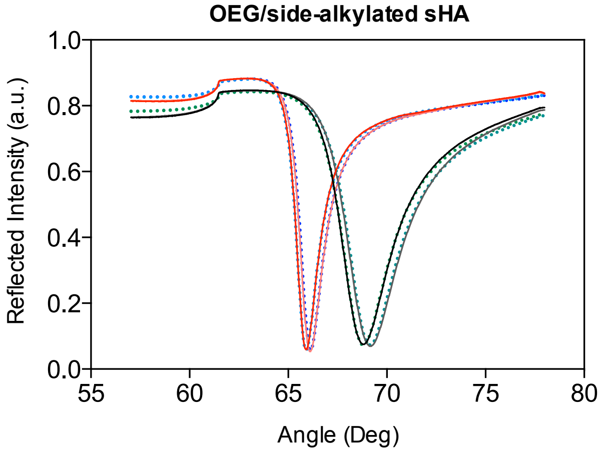

Figure S5. Example fit of OEG/side-alkylated sHA layer (curves represent before and after sample deposition) using BioNavis LayerSolver. Red and black lines show the measured data at 785 and 670 nm respectively. Modeled data represented by blue (785 nm) and green (670 nm) dotted lines.

Control experiment for the adsorption of aggrecan and Lyve-1+Ab on EG_3OH adlayer. We investigated if aggrecan and Lyve-1+Ab complex would interact unspecifically with the oligo(ethylene) glycol layer when it is used to adjust the density of sHA. However, both aggrecan and Lyve-1+Ab do not induce any frequency or significant dissipation change on the EG_3OH layer indicating that the surface is inert against aggrecan and Lyve1+Ab binding.

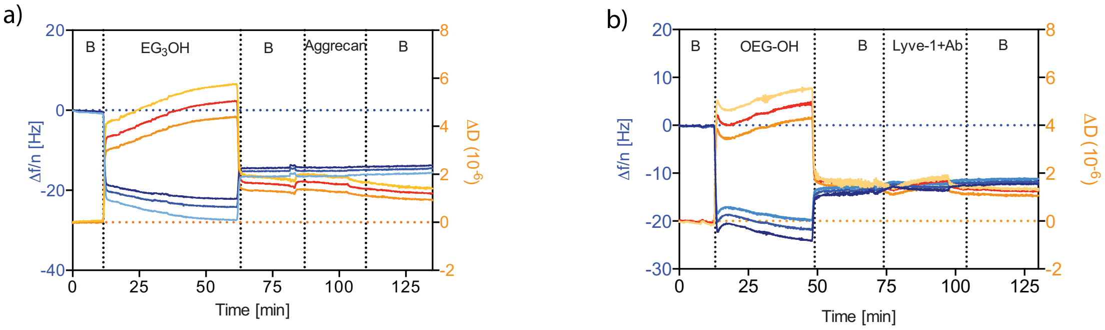

Figure S6. (a) Aggrecan (100 μg/mL) and (b) Lyve-1+Ab do not bind to EG_3OH (100 μM) adlayers.

Comparison of the induced dissipation changes on sHA layers with different grafting densities. Aggrecan binding was also probed at different densities of OEG/side&end-alkylated sHA. The results indicate qualitatively that a slight decrease in dissipation change was observed when OEG/side&end-alkylated sHA density is lowered. In addition, the highest dissipation change was observed when the eSH-sHA high-density coverage was used.

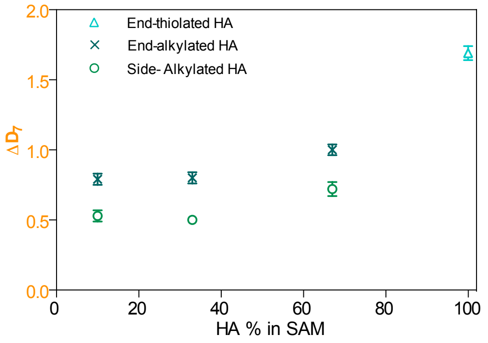

Figure S7. Comparison between the dissipation change vs. sHA density in the adlayer.

Evaluation of Lyve-1+Antibody binding to side and end-alkylated sHA. The semi-quantitative evaluation of the frequency and dissipation changes upon Lyve-1+Antibody and antibody binding on the side and end–alkylated surfaces are shown in Figure S8. As seen from the graphs, there is an apparent difference especially Lyve-1+Antibody vs antibody binding on these surfaces.

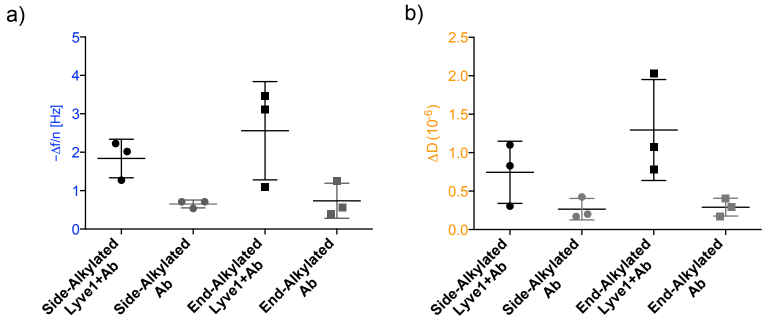

Figure S8. Scatter plots (with mean and standard deviation) show the (a) frequency and (b) dissipation change upon Lyve-1+antibody complex vs antibody binding on the end and side alkylated sHA surfaces.

References

1 Gutig, C., Grady, B. P. & Striolo, A. Experimental studies on the adsorption of two surfactants on solid-aqueous interfaces: adsorption isotherms and kinetics. *Langmuir* **24**, 4806-4816 (2008).

2 Berts, I., Fragneto, G., Hilborn, J. & Rennie, A. R. Tuning the density profile of surface-grafted hyaluronan and the effect of counter-ions. *Eur. Phys. J. E Soft Matter* **36**, 70 (2013).

3 Albers, W. & Vikholm-Lundin, I. in *Nano-Bio-Sensing* (ed Sandro Carrara) Ch. 4, 83-125 (Springer New York, 2011).
